# Association between socio-economic status and hemoglobin A1c levels in a Canadian primary care adult population without diabetes

**DOI:** 10.1186/1471-2296-15-7

**Published:** 2014-01-10

**Authors:** Babak Aliarzadeh, Michelle Greiver, Rahim Moineddin, Christopher Meaney, David White, Ambreen Moazzam, Kieran M Moore, Paul Belanger

**Affiliations:** 1 Department of Family and Community Medicine, University of Toronto, 500 University Avenue, 5th Floor, Toronto M5G 1 V7, ON, Canada; 2 Public Health Informatics Group, Kingston, Frontenac, Lennox & Addington Public Health, 221 Portsmouth Avenue, Kingston, ON K7M 1 V5, Canada; 3 Department of Emergency Medicine, Queen’s University, Kingston, ON K7L 3 N6, Canada; 4 North York General Hospital, 4001 Leslie St, Toronto, ON M2K 1E1, Canada

## Abstract

**Background:**

Hgb A1c levels may be higher in persons without diabetes of lower socio-economic status (SES) but evidence about this association is limited; there is therefore uncertainty about the inclusion of SES in clinical decision support tools informing the provision and frequency of Hgb A1c tests to screen for diabetes. We studied the association between neighborhood-level SES and Hgb A1c in a primary care population without diabetes.

**Methods:**

This is a retrospective study using data routinely collected in the electronic medical records (EMRs) of forty six community-based family physicians in Toronto, Ontario. We analysed records from 4,870 patients without diabetes, age 45 and over, with at least one clinical encounter between January 1st 2009 and December 31st 2011 and one or more Hgb A1c report present in their chart during that time interval. Residential postal codes were used to assign neighborhood deprivation indices and income levels by quintiles. Covariates included elements known to be associated with an increase in the risk of incident diabetes: age, gender, family history of diabetes, body mass index, blood pressure, LDL cholesterol, HDL cholesterol, triglycerides, and fasting blood glucose.

**Results:**

The difference in mean Hgb A1c between highest and lowest income quintiles was -0.04% (p = 0.005, 95% CI -0.07% to -0.01%), and between least deprived and most deprived was -0.05% (p = 0.003, 95% CI -0.09% to -0.02%) for material deprivation and 0.02% (p = 0.2, 95% CI -0.06% to 0.01%) for social deprivation. After adjustment for covariates, a marginally statistically significant difference in Hgb A1c between highest and lowest SES quintile (p = 0.04) remained in the material deprivation model, but not in the other models.

**Conclusions:**

We found a small inverse relationship between Hgb A1c and the material aspects of SES; this was largely attenuated once we adjusted for diabetes risk factors, indicating that an independent contribution of SES to increasing Hgb A1c may be limited. This study does not support the inclusion of SES in clinical decision support tools that inform the use of Hgb A1c for diabetes screening.

## Background

Hemoglobin A1c (Hgb A1c) represents an average blood glucose over three months [1,2]. It is recommended as a screening measure and diagnostic test for diabetes [3-5]. Increasing levels of Hgb A1c are strongly associated with greater risk of incident diabetes; Hgb A1c levels of 6% or more lead to a five year risk of diabetes ranging from 25% to 50% [6].

Lower socio-economic status (SES) is associated with higher rates of mortality and morbidity [7-9]. The Deprivation Index is a method of measuring neighborhood level SES. The index includes a material dimension (referring to the ability to obtain goods and services) and a social dimension (referring to connections with families, communities and workplaces) [9]. Both material and social deprivation have been associated with higher mortality rates across Canada [9].

Lower SES levels may be associated with a higher risk of incident diabetes, especially amongst women [10-15]. Previous studies suggest that levels of Hgb A1c in persons without diabetes may increase as socio-economic deprivation worsens [2,11,16], leading to the hypothesis that stress associated with deprivation may have an effect on glycemic control [2]. Most published studies in this area have used surveys or data collected for randomized controlled trials done for other purposes [2,11,16]. For example, a cross sectional survey assessed the association between SES and Hgb A1c levels in 1,828 persons without diabetes, controlling for fasting glucose levels, age and alcohol consumption [16]. Lower family income and lower educational level were both associated with increasing Hgb A1c levels. A sub-analysis conducted within a randomized controlled trial (the Women’s Health Study) found that, for professional women, there was an association between lower income and lower educational level and risk of incident diabetes. The risk was attenuated after adjustment for cardiovascular risk factors [11]. In another study, lower grade of employment (clerical versus professional) was associated with higher Hbg A1c levels in British civil servants [2].

Recent research [17,18] and guidelines [5] have recommended that diabetes risk stratification using validated calculators be used to guide the provision and frequency of diabetes screening. Patients at low risk may not need to be screened; those at very high risk should be screened annually with Hgb A1c [5]. Factors included in validated calculators recommended in Canada [5,17,18] include age, gender, body mass index, previous abnormal glucose levels, hypertension, family history of diabetes, lack of exercise, and low intake of fruits and Vegetables [5].

A systematic review found seven risk models thought to be potentially adaptable for routine clinical practice [19]. Only one risk model, the QD Score from the UK [20], included SES as a risk factor [19]. There is currently insufficient evidence to support the inclusion or exclusion of SES as an independent risk factor in clinical decision support calculators guiding the provision of Hgb A1c to screen for diabetes.

Routinely collected primary care data had not been easily available in the past, and has not been used to investigate associations between SES and Hgb A1c. As well, the Deprivation Index, which aggregates several aspects of SES, has not yet been used to study Hgb A1c levels in persons without diabetes. Electronic Medical Records (EMRs) present a rich new source of primary care data. Using a Practice Based Research Network EMR database, we previously found that the use of screening Hgb A1c in persons without diabetes in Toronto, Ontario had been rising [21]. In that study one fifth of all patients without diabetes, age 45 or over, had a Hgb A1c test done in the three years prior to the release of guideline recommendations to use this test in January 2010. Patients tested were more likely to have risk factors for incident diabetes (higher fasting blood glucose, higher body mass index, older age, or presence of hypertension) than those not tested. As part of that study, we examined the association between area level SES and the provision of Hgb A1c testing. We found no difference in the adjusted odds ratio of having the test done by SES quintile other than for the highest income quintile, which had lower adjusted odds ratio than the lowest quintile.

For the current study, we examined the association between neighborhood level income as well as the social and material dimensions of deprivation and Hgb A1c levels in a primary care population without diabetes. In addition to testing the overall association of Deprivation Indices and income with Hgb A1c levels, we studied the relationship after adjustment for several factors associated with an increased risk of incident diabetes [3,4], body mass index, blood pressure, fasting blood glucose, dyslipidemia [22], age, gender and history of diabetes in blood relatives [19]. The objective of this study was to examine the association between SES and Hgb A1c levels in persons without diabetes. We tested the hypothesis that decreasing neighborhood SES level is associated with increasing Hgb A1c, and that this persists after controlling for factors predictive of an increased risk of incident diabetes.

## Methods

### Participants

Data from forty-six primary care providers in Toronto, Canada, participating in the Canadian Primary Care Sentinel Surveillance Network (CPCSSN) as part of the University of Toronto Practice Based Research Network (UTOPIAN) were used for this study. Forty two of these providers practice within interdisciplinary primary care models. CPCSSN is Canada's first multi-disease Electronic Medical Record (EMR) based surveillance system [23]. UTOPIAN is one of ten networks currently participating in CPCSSN and is a primary care practice based research network affiliated with the Department of Family and Community Medicine at the University of Toronto. Family physicians participating in UTOPIAN can contribute anonymized EMR data to the local CPCSSN repository; data from all participating networks are aggregated in a single national database. Posters informing patients about the study are present in the waiting rooms of participating practices, and patients can opt out [23]. Patients were formally enrolled with their physician, allowing the identification of practice panels [24]. Data were extracted from three different EMR software applications (Nightingale-on-Demand®, Practice Solutions® and Bell EMR®).

The eligible population was comprised of patients age 45 and over, who were active and enrolled with a participating physician as of December 31^st^ 2011. We included patients who had at least one encounter in the EMR between January 1^st^ 2009 and December 31^st^ 2011. We determined diabetes status using the same methods as our previous study [21]; only patients without diabetes with at least one measurement of Hgb A1c during the three years of interest were included. We excluded patients with sickle cell anemia or other hemoglobinopathies as indicated in their health profiles; these conditions are known to affect Hgb A1c [1,25]. Patients with missing covariate data were also excluded.

### Data sources

The following data elements were extracted from the CPCSSN database: body mass index (BMI), systolic and diastolic blood pressures, LDL and HDL cholesterol, triglycerides(TG), fasting blood glucose (FBG), age in years (at time of Hgb A1c measurement), gender, and Hgb A1c. If multiple measurements of Hgb A1c were present, we used the last Hgb A1c; covariates measured concurrent with the last Hgb A1c test or at the closest date were used in the analysis. Family history was not available in the CPCSSN database; data on family history of diabetes were directly extracted from the EMR databases.

We used geographically derived information to calculate income level as well as social and material deprivation indices [9]. The Postal Code Conversion File, available from Statistics Canada, was used to link the six-character postal codes to the standard 2006 Census dissemination areas. Dissemination areas are small, stable parts of neighborhoods that include between 400 and 700 persons [26]. Subsequently, the Postal Code Conversion File was used to assign neighborhood income and deprivation indices (updated for 2006 census) [27] to the patients' residential postal codes. A detailed description and method for calculating these indices have been published elsewhere [9]. In summary, Pampalon and Raymond used six neighborhood level indicators, namely: 1) proportion of people who have not graduated from high school, 2) ratio of employment to population, 3) mean income, 4) proportion of persons who are separated, divorced or widowed, 5) proportion of single-parent families; and 6) proportion of people living alone. The first three indicators were combined and were used to determine the "material" aspects of deprivation by quintiles; the last three were used to determine the "social" aspects of deprivation by quintiles. These indices are available at the geographic dissemination area level.

### Statistical analysis

Following previous research on neighbourhood characteristics and individual patient factors [8,28,29], we examined the adequacy of a hierarchical analysis that takes into account the lack of independence of data (geographical clustering) to analyse data. Hierarchical models reduce to non-hierarchical analysis when the variation at higher level is not significant; this was the case in our study. We used type 3 likelihood based F-statistics from a linear mixed model analysis with random intercept to examine the association of SES quintiles with increasing Hgb A1c in each model. We determined bivariate associations with Hgb A1c as outcome using a random intercept linear mixed model. We added the SES variables to the linear mixed model at the area level (level 2) and all other variables under consideration were added at the patient level (level 1). We compared mean Hgb A1c for the least deprived and the most deprived quintiles for each SES model, as well as for other variables of interest, using the likelihood ratio test. Regression models were adjusted for covariates found to increase the risk of incident diabetes in bivariate analyses [28]. We repeated our analysis for the adjusted models; we did not include fasting blood glucose in the adjusted models due to the correlation between fasting blood glucose and Hgb A1c [30,31]. P values of less than 0.05 were considered significant.

Data were analysed using SAS version 9.3. The North York General Hospital Research Ethics Board reviewed and approved the study.

## Results

**Figure 1:**
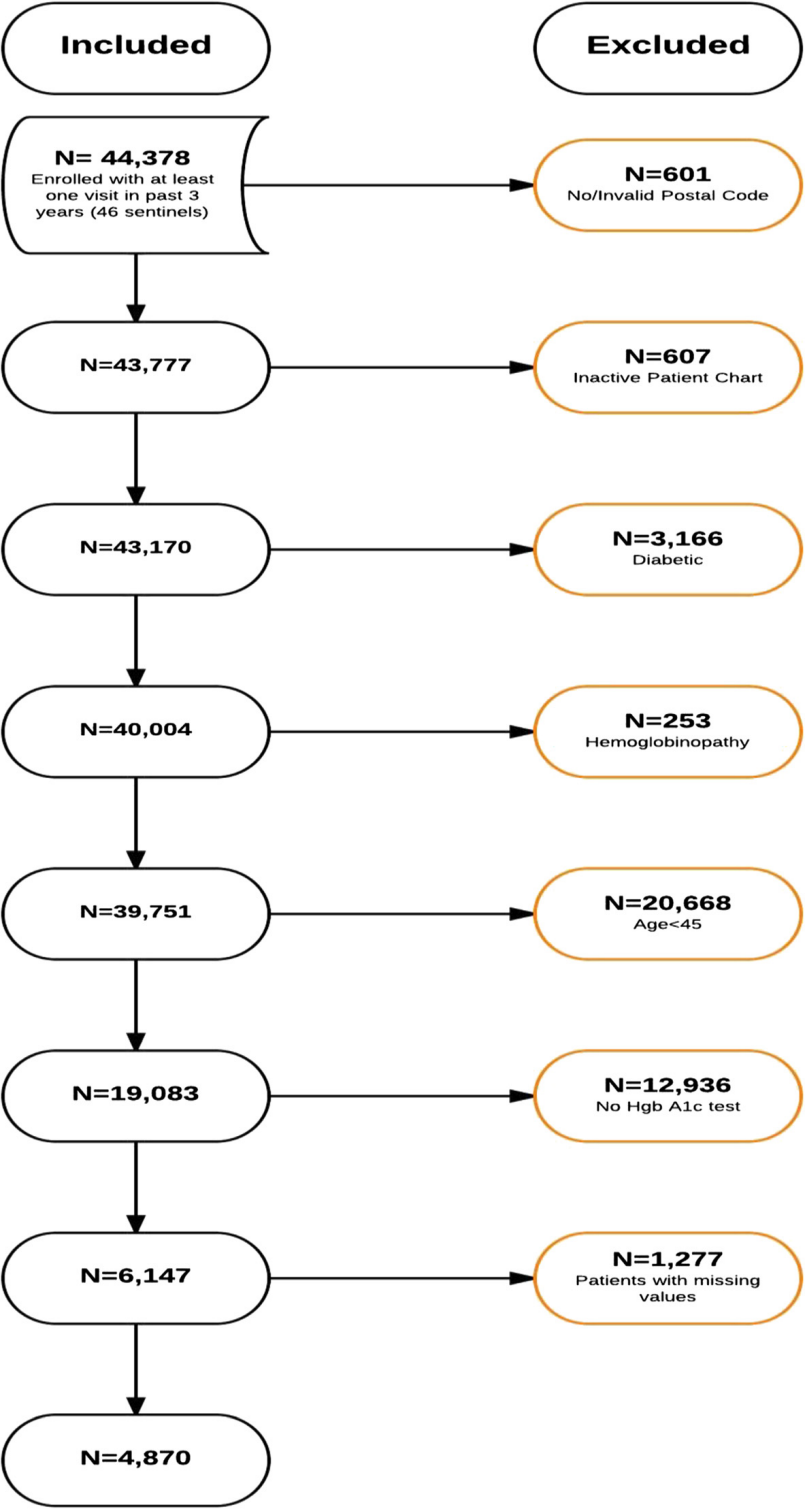
Flow diagram detailing steps followed in the generation of the study sample.

There were 19,083 eligible patients age 45 or more; 6,147 (32%) had a screening Hgb A1c measured at least once in the three years of interest. Patients with missing covariates were removed from the analysis. This resulted in a final sample of 4,870 patients. A flow diagram detailing the steps followed in the generation of the study sample is shown in Figure 1.

The distribution of Hgb A1c in this sample was roughly Gaussian. Table 1 presents patient characteristics.

**Table 1:** **Characteristics of the 4**,**870 non-diabetic patients included in the analysis**

**Characteristic (unit)**	**Mean (±SD) or N (%)**
Hgb A1c (%)	5.7 (±0.4)
LDL (mmol/L)	3.2 (±0.9)
HDL(mmol/L)	1.5 (±0.4)
Cholesterol (mmol/L)	5.3 (±1.0)
TG (mmol/L)	1.3 (±0.8)
Weight (kg)	75.9 (±18.1)
Obese	1413 (29.0%)
BMI (kg/m^2)	27.5 (±5.1)
SBP (mmHg)	126.1 (±17.2)
DBP (mmHg)	77.3 (±9.8)
FBG (mmol/L)	5.3 (±0.6)
FBG 6–6.9 (mmol/L)	723 (14.8%)
Age (years)	62.5 (±11.2)
Male	1834 (37.7%)
Hypertension	1981 (40.7%)
Family history of diabetes	909 (18.7%)
Income (Quintile)	
Lowest income (Q1)	914 (18.8%)
Low income (Q2)	765 (15.7%)
Mid income (Q3)	868 (17.8%)
High income (Q4)	914 (18.8%)
Highest income (Q5)	1409 (28.9%)
Material deprivation (Quintile)	
Most deprived (Q5)	549 (11.3%)
Deprived (Q4)	729 (15.0%)
Neutral (Q3)	914 (18.8%)
Less deprived (Q2)	1193 (24.5%)
Least deprived (Q1)	1485 (30.5%)
Social deprivation (Quintile)	
Most deprived (Q5)	981 (20.1%)
Deprived (Q4)	1147 (23.5%)
Neutral (Q3)	985 (20.2%)
Less deprived (Q2)	905 (18.6%)
Least deprived (Q1)	852 (17.5%)
Combined material and social deprivation (Quintile)	
Most deprived (Q5)	605 (12.4%)
Deprived (Q4)	963 (19.8%)
Neutral (Q3)	1087 (22.3%)
Less deprived (Q2)	1076 (22.1%)
Least deprived (Q1)	1139 (23.4%)

SD—standard deviation, LDL—low density lipoprotein, HDL – high density lipoprotein, TG—triglycerides, FBG—fasting blood glucose, BMI – body mass index, SBP—systolic blood pressure, DBP—diastolic blood pressure, Q1—first quintile, Q2—second quintile, Q3—third quintile, Q4—fourth quintile, Q5—fifth quintile.

There were 1,852 distinct dissemination areas in the sample. Our sample ranged from 1 person to 35 persons per dissemination area. The variation of our Hgb A1c outcome at the dissemination area level (level 2) was not large or statistically significant. The estimated intraclass correlation coefficient (ICC) for the Hgb A1c outcome within dissemination areas was 0.005 (p = 0.3).

Table 2 presents unadjusted estimates for Hgb A1c across SES quintiles. Using a type 3 likelihood based F-statistics from a linear mixed model analysis with random intercept, we found a statistically significant association between decreasing income quintile (p = 0.0005) and increasing Hgb A1c. There was a similar association for worsening material deprivation (p = 0.02). There was no statistically significant association between increasing Hgb A1c and social deprivation (p = 0.3) or combined deprivation index (p = 0.7).

**Table 2:** Mean and standard deviation for Hgb A1c by quintile of SES attribute

**SES attribute**	**Least deprived/Highest income**	**Less deprived/High income**	**Neutral/Mid income**	**Deprived/Low income**	**Most deprived/Lowest income**
	**Mean (SD)**	**Mean (SD)**	**Mean (SD)**	**Mean (SD)**	**Mean (SD)**
Income	5.68 (0.38)	5.68 (0.38)	5.69 (0.35)	5.72 (0.37)	5.72 (0.37)
Material deprivation	5.68 (0.34)	5.69 (0.37)	5.71 (0.37)	5.68 (0.35)	5.74 (0.38)
Social deprivation	5.70 (0.34)	5.68 (0.37)	5.70 (0.37)	5.71 (0.35)	5.68 (0.36)
Material and social deprivation	5.69 (0.34)	5.69 (0.37)	5.69 (0.36)	5.70 (0.36)	5.71 (0.38)

SES = Socio-economic status.

SD = Standard Deviation.

Table 3 presents bivariate results for comparisons of differences in mean Hgb A1c. There were statistically significant associations between increasing Hgb A1c levels and dyslipidemia, increasing BMI, increasing blood pressure, increasing fasting blood glucose, increasing age, female gender and presence of a family history of diabetes.

**Table 3:** Bivariate results from a random intercept Linear Mixed Model comparing mean Hgb A1c levels

	**Difference in mean Hgb A1c (95% CI)**	**P-value**
Income (Quintile)		
• Lowest income (Q1) (Reference)	---	---
• Low income (Q2)	-0.00 (-0.04 to 0.03)	0.9
• Mid income (Q3)	-0.03 (-0.06 to 0.003)	0.07
• High income (Q4)	-0.04 (-0.08 to 0.01)	0.01
• Highest income (Q5)	-0.04 (-0.07 to 0.01)	0.005
Material deprivation (Quintile)		
• Most deprived (Q5) (Reference)	---	---
• Deprived (Q4)	-0.05 (-0.09 to 0.01)	0.008
• Neutral (Q3)	-0.03 (-0.07 to 0.01)	0.1
• Less deprived (Q2)	-0.05 (-0.09 to 0.01)	0.005
• Least deprived (Q1)	-0.05 (-0.09 to 0.02)	0.003
Social deprivation (Quintile)		
• Most deprived (Q5) (Reference)	---	---
• Deprived (Q4)	0.03 (-0.001 to 0.06)	0.06
• Neutral (Q3)	0.02 (-0.01to 0.05)	0.3
• Less deprived (Q2)	0.00 (-0.03 to 0.04)	0.8
• Least deprived (Q1)	0.02 (-0.01 to 0.06)	0.2
Combined material and social deprivation (Quintile)		
• Most deprived (Q5) (Reference)	---	---
• Deprived (Q4)	-0.01(-0.05 to 0.02)	0.5
• Neutral (Q3)	-0.02 (-0.06 to 0.02)	0.3
• Less deprived (Q2)	-0.02 (-0.06 to 0.02)	0.3
• Least deprived (Q1)	-0.02 (-0.06 to 0.01)	0.2
LDL*	0.02 (0.01 to 0.03)	0.0002
HDL*	-0.07 (-0.09 to 0.05)	<0.0001
Triglycerides*	0.04 (0.03 to 0.05)	<0.0001
Obese†	0.07 (0.04 to 0.09)	<0.0001
BMI‡	0.01 (0.004 to 0.01)	<0.0001
SBP **	0.00 (0.001 to 0.003)	<0.0001
DBP **	0.00 (0.01 to 0.002)	0.0005
FBG*	0.14 (0.12 to 0.16)	<0.0001
FBG 6–6.9	0.17 (0.13 to 0.01)	<0.0001
Age§	0.01 (0.005 to 0.008)	<0.0001
Male	-0.02 (-0.04 to -0.01)	0.04
Family History of Diabetes	0.03 (0.002 to 0.05)	0.03

LDL—low density lipoprotein, HDL – high density lipoprotein, FBG—fasting blood glucose, BMI – body mass index, SBP—systolic blood pressure, DBP—diastolic blood pressure, Q1—first quintile, Q2—second quintile, Q3—third quintile, Q4—fourth quintile, Q5—fifth quintile.

* for each additional 1 mmol/L.

† BMI 30 or more.

** mmHg.

‡ for each additional 1 unit.

§ for each additional year.

The difference in mean Hgb A1c between highest and lowest income quintiles was -0.04% (p = 0.005). The difference between least deprived and most deprived was -0.05% (p = 0.003) for material deprivation, 0.02% (p = 0.2) for social deprivation, and -0.02% (p = 0.2) for combined material and social deprivation.

Table 4 presents the results of a multivariate analysis in four separate SES models, after adjusting for the statistically significant variables in the bivariate analysis, with the exclusion of fasting blood glucose. There was a statistically significant difference in mean Hgb A1c levels between the most and least deprived (p = 0.04), less deprived (p = 0.03), and deprived (p = 0.02) quintiles in the material deprivation model, but not in any of the other SES models. After adjustment, there were statistically significant associations between increasing Hgb A1c levels and increasing LDL, decreasing HDL, increasing TG, increasing age and female gender in all models. There were no significant associations with Hgb A1c for increasing systolic or diastolic blood pressures in any model.

**Table 4:** Results of multivariate linear mixed models estimating association between Hgb A1c levels, SES and associated variables across four different models

	**Income model**		**Material deprivation model**		**Social deprivation model**		**Combined material and social deprivation model**	
	**Difference in mean Hgb A1c (95% CI)**	**P-value**	**Difference in mean Hgb A1c (95% CI)**	**P-value**	**Difference in mean Hgb A1c (95% CI)**	**P-value**	**Difference in mean Hgb A1c (95% CI)**	**P-value**
Deprivation								
• Most deprived (reference)	---	---	---	---	---	---	---	---
• Deprived	0.004 (-0.028 to 0.038)	0.78	-0.047 (-0.085 to -0.008)	0.02	0.019 (-0.011 to 0.048)	0.21	-0.017 (-0.052 to 0.018)	0.34
• Neutral	-0.019 (-0.052 to 0.012)	0.23	-0.029 (-0.065 to 0.007)	0.12	0.014 (-0.016 to 0.044)	0.37	-0.011 (-0.046 to 0.023)	0.52
• Less deprived	-0.023 (-0.055 to 0.008)	0.15	-0.040 (-0.075 to -0.004)	0.03	0.008 (-0.023 to 0.039)	0.62	-0.004 (-0.039 to 0.030)	0.79
• Least deprived	-0.020 (-0.049 to 0.009)	0.18	-0.035 (-0.069 to -0.001)	0.04	0.022 (-0.009 to 0.054)	0.18	-0.010 (-0.044 to 0.024)	0.57
LDL*	0.021 (0.010 to 0.032)	0.0001	0.020 (0.01 to 0.031)	0.0002	0.0214 (0.011 to 0.032)	0.0001	0.021 (0.010 to 0.032)	0.0001
HDL*	-0.067 (-0.094 to -0.039)	<0.0001	-0.068 (-0.095 to -0.040)	<0.0001	-0.067 (-0.095 to 0.040)	<0.0001	-0.068 (-0.09 to -0.040)	<0.0001
Triglycerides*	0.021 (0.008 to 0.034)	0.001	0.021 (0.008 to 0.033)	0.001	0.021 (0.008 to 0.034)	0.001	0.021 (0.008 to 0.034)	0.001
BMI†	0.004 (0.002 to 0.006)	<0.0001	0.004 (0.002 to 0.006)	<0.0001	0.004 (0.002 to 0.006)	<0.0001	0.004 (0.002 to 0.006)	<0.0001
SBP‡	0.003 (-0.001 to 0.001)	0.53	0.001 (-0.001 to 0.001)	0.59	0.001 (-0.001 to 0.001)	0.54	0.001 (-0.001 to 0.001)	0.55
DBP‡	0.001 (-0.001 to 0.002)	0.20	0.001 (-0.001 to 0.002)	0.17	0.001 (-0.001 to 0.002)	0.20	0.001 (-0.001 to 0.002)	0.19
Age§	0.007 (0.006 to 0.008)	<0.0001	0.007 (0.006 to 0.008)	<0.0001	0.007 (0.006 to 0.008)	<0.0001	0.001 ( 0.006 to 0.008)	<0.0001
Male gender	-0.050 (-0.073 to -0.028)	<0.0001	-0.051 (-0.074 to -0.029)	<0.0001	-0.051 (-0.073 to -0.028)	<0.0001	-0.051 (-0.073 to -0.029)	<0.0001
Family History of Diabetes	0.030 (0.005 to 0.055)	0.02	0.031 (0.005 to 0.055)	0.02	0.04 (0.005 to 0.056)	0.02	0.031 ( 0.006 to 0.056)	0.02

LDL—low density lipoprotein, HDL – high density lipoprotein, BMI – body mass index, SBP—systolic blood pressure, DBP—diastolic blood pressure.

* For each additional 1 mmol/L.

† For each additional 1 unit.

‡ For each additional 1 mmHg. § for each additional year.

To test the reliability of our results, we conducted several sensitivity analyses. Re-analysis of data using data from all 6,147 patients with a Hgb A1c present led to the same conclusions. We added fasting blood glucose (FBG) to the multivariate models; this eliminated the marginal difference in the material deprivation model. As a result, there was no significant difference in Hgb A1c between highest and lowest SES quintile in any model. We also replaced systolic and diastolic blood pressures with history of hypertension (yes/no). None of the hypertension measures were statistically significant in the multivariate models, and the overall conclusion did not change.

## Discussion

We studied the association of Hgb A1c levels with annual income, material and social deprivation in a Canadian primary care population. We found an inverse relationship between SES and Hgb A1c and very small differences in mean Hgb A1c levels between the most materially deprived populations and those with lesser deprivation. A threshold for clinically significant differences in Hgb A1c in the Canadian setting has been agreed upon as being 10% [32]. In our study, this would have meant differences in Hgb A1c of 0.6% or more; we found differences that were an order of magnitude smaller. These differences were no longer statistically significant in three out of four SES models after adjustment for other factors known to be associated with an increase in the incidence of diabetes.

Previous studies have found an association between lower income, lower education level, lower employment grade and increasing Hgb A1c in persons without diabetes [2,11,16]. A strength of this study is the fact that we were able to study a larger primary care sample, and adjusted for multiple factors associated with a greater risk of incident diabetes. As well, we used validated indices aggregating several aspects of deprivation.

The literature was unclear as to which elements of deprivation might be associated with Hgb A1c levels; we therefore used several validated indices of deprivation (income, material, social, combined). This is similar to the approach taken in a recent study to study obesity and deprivation [28]. After adjustment, only material deprivation was statistically associated with Hgb A1c levels, although the difference was small and not clinically significant.

Neighborhood deprivation may be associated with increases in cardiometabolic risk factors and levels of obesity [28,33,34]. Several of these risk factors are associated with an increased risk of incident diabetes [3,19]. These could explain the higher rates of incident diabetes observed in deprived areas [13,14], rather than neighborhood poverty independently affecting Hgb A1c.

We found associations between increasing Hgb A1c and factors included in diabetes risk calculators [17-19], such as increasing BMI, increasing blood pressure, increasing fasting blood glucose, age, gender, family history and dyslipidemia. This supports the use of these factors to assist in diabetes screening decisions.

We also noted an increase in the uptake of Hgb A1c screening in patients age 45 or more, from 20% in our previous study (immediately prior to the release of guidelines recommending this test in persons at risk of developing diabetes) [21] to 32% in the current study, two years after the release of the new guidelines.

In the Canadian context, citizens have universal coverage for health care. The finding that lower SES was not an independent risk factor for elevated Hgb A1c in the patients of family physicians in this study may be generalizable to clinical settings where SES is not a barrier to accessing primary care. However, this lack of association should not be interpreted to mean that interventions to reduce the risk of diabetes in lower SES neighbourhoods are inapplicable at the population level.

### Limitations

We used an ecological approach to measure deprivation as we could not determine deprivation directly at the individual level. However, the measures we used have been validated and have been extensively employed in other studies.

We had several data limitations. Waist circumference is an important predictor of diabetes. We could not include this factor as 75% of our sample population did not have a waist circumference recorded in the EMR during the three years of interest. Some of the records lacked precision as to which relative had a history of diabetes; in other words, we did not know whether the family history was in a first degree relative. For this study, family history of diabetes was defined as having a recorded history of diabetes in any blood relative. Ethnicity is an important predictor of diabetes [35,36], but is poorly captured in the EMRs we used and could not be included. Similarly, EMR data on physical activity levels, smoking history, and diet are incomplete, and could not be used in this study. Postal code was missing for a small percentage of patients(less than 2%); while some of these patients may be homeless, the proportion is not large enough to invalidate our study results. Only 37.7% of the sample was male. Male patients represent 36% of the CPCSSN patient population in Toronto; the percentage of males in study population was similar to that of the source primary care population [37].

This was an observational study using EMR data, and there were likely systematic differences between patients tested and not tested using Hgb A1c that could impact the generalizability, but not internal validity, of these findings. In our previous study, patients with factors associated with a higher risk of incident diabetes were more likely to have the test done [21]. As well, persons living in the lowest income quintile neighborhoods had a higher adjusted odds ratio of having a screening Hgb A1c test done than those living in the highest income quintile (OR 0.63) [21]. However, selectively testing patients with more risk factors, which tend to cluster in poorer neighborhoods, may lead to bias towards falsely positive differences instead of our generally negative results. Lastly, our study reflects conditions for persons living in a largely urban setting in southern Ontario; factors affecting neighborhood deprivation may differ in other settings.

## Conclusions

We found a small, inverse relationship between SES and Hgb A1c; this was not clinically significant. Lower SES was not an independent predictor of higher Hgb A1c in three out of four models, after adjusting for covariates associated with an increased risk of incident diabetes. This study does not support the inclusion of SES levels as an additional independent risk factor in clinical decision support tools informing diabetes screening with Hgb A1c in this primary care setting.

Postal codes were available in 99% of records we extracted from the EMRs. Given the limited extent and quality of SES data available in clinical records, an ecologic approach as used in this study appears to be a feasible method of examining deprivation in Canadian EMR databases. This could be used in future research on chronic disease management and prevention as well as for program planning that addresses local population needs in primary care settings.

## Abbreviations

Hgb A1c: Hemoglobin A1c; SES: Socio-economic status; CPCSSN: Canadian primary care sentinel surveillance network; UTOPIAN: University of Toronto practice based research network; EMR: Electronic Medical record; BMI: Body mass index; TG: Triglycerides; FBG: Fasting blood glucose.

## Competing interests

The authors declare that they have no competing interests.

## Authors' contributions

BA and MG conceived the study. All authors participated in the design of the study. BA performed the data extraction and preparation for analysis. RM and CM performed the statistical analyses. BA, MG, RM, CM, DW and AM participated in the coordination of the study. All authors participated in the interpretation of study results, drafting of the manuscript, and read and approved the final manuscript.

## Pre-publication history

The pre-publication history for this paper can be accessed here:

http://www.biomedcentral.com/1471-2296/15/7/prepub
